# The neuroprotective effects of activated α7 nicotinic acetylcholine receptor against mutant copper–zinc superoxide dismutase 1-mediated toxicity

**DOI:** 10.1038/s41598-020-79189-y

**Published:** 2020-12-17

**Authors:** Taisei Ito, Masatoshi Inden, Tomoyuki Ueda, Yuta Asaka, Hisaka Kurita, Isao Hozumi

**Affiliations:** Laboratory of Medical Therapeutics and Molecular Therapeutics, Gifu Pharmaceutical University, 1-25-4 Daigaku-nishi, 1-1-1, Gifu, 501-1196 Japan

**Keywords:** Cell death in the nervous system, Molecular neuroscience

## Abstract

Amyotrophic lateral sclerosis (ALS) is a fatal neurodegenerative disease characterized by the selective and progressive loss of motor neurons. Although many drugs have entered clinical trials, few have shown effectiveness in the treatment of ALS. Other studies have shown that the stimulation of α7 nicotinic acetylcholine receptor (nAChR) can have neuroprotective effects in some models of neurodegenerative disease, as well as prevent glutamate-induced motor neuronal death. However, the effect of α7 nAChR agonists on ALS-associated mutant copper–zinc superoxide dismutase 1 (SOD1) aggregates in motor neurons remains unclear. In the present study, we examined whether α7 nAChR activation had a neuroprotective effect against SOD1^G85R^-induced toxicity in a cellular model for ALS. We found that α7 nAChR activation by PNU282987, a selective agonist of α7 nAChR, exhibited significant neuroprotective effects against SOD1^G85R^-induced toxicity via the reduction of intracellular protein aggregates. This reduction also correlated with the activation of autophagy through the AMP-activated protein kinase (AMPK)–mammalian target of rapamycin (mTOR) signaling pathway. Furthermore, the activation of α7 nAChRs was found to increase the biogenesis of lysosomes by inducing translocation of the transcription factor EB (TFEB) into the nucleus. These results support the therapeutic potential of α7 nAChR activation in diseases that are characterized by SOD1^G85R^ aggregates, such as ALS.

## Introduction

Amyotrophic lateral sclerosis (ALS) is an adult-onset neurological disorder that is characterized by muscle weakness and atrophy, paralysis, and eventual death by respiratory failure. Symptoms result from the selective degeneration of upper and lower motor neurons. While 90–95% of ALS cases arise sporadically, 5–10% are familial in nature. In previous studies searching for causal genes associated with familial ALS, copper–zinc superoxide dismutase 1 (*SOD1*), TAR DNA binding protein of 43 kDa (*TDP43*), fused in sarcoma (*FUS*), and optineurin (*OPTN*) have been identified as playing roles in pathological cascades and phase transitions in sporadic ALS^1–7^. Among familial ALS patients, mutation in SOD1 is a major autosomal dominant inherited allele associated with disease^8^.

Several clinical studies suggest a role for glutamate-induced excitotoxicity in familial ALS pathology, although the potential pathogenesis in sporadic ALS is still unclear^9–11^. Previous experiments have shown that rat spinal cord cultures exposed to long-term low-dose glutamate exhibit selective motor neuronal death^12–14^. For this reason, riluzole, a glutamate release inhibitor, has been developed as a therapeutic agent for ALS patients, and has been shown to prolong patient life by several months^15^. Although many drugs have entered into clinical trials, few have demonstrated any effectiveness in the treatment of ALS. Therefore, new prospective drugs are strongly desired in the clinical field.

Nine α (α2–α10) and three β (β2–β4) nicotinic acetylcholine receptor (nAChR) subunits are expressed in the vertebrate brain^16^. These subunits coassemble to form a family of functionally diverse nAChRs. Among nAChR subunits, the most abundant subtypes in the mammalian nervous system are homomeric α7 nAChRs and heteromeric β2 nAChRs, including α4β2 nAChRs^16–19^. These receptors are also widely expressed in the dorsal and ventral horns in the spinal cord^14,20–22^. Loss of nAChRs is associated with a number of disease states, including Alzheimer’s disease, Parkinson’s disease (PD), Lewy body disease, schizophrenia, autism, and attention deficit/hyperactivity disorder (ADHD)^23^. In addition, accumulating evidence suggests that α7 nAChRs are important targets in the development of therapeutics for PD. We have previously demonstrated that α7 nAChR activation protects against dopaminergic neuronal death in both acute and chronic animal models of PD induced by 6-hydroxydopamine (6-OHDA) and rotenone, respectively^24,25^.

Nagano et al. have also reported an early decrease in cholinergic input in motor neurons in the spinal cords of patients with ALS^26^. nAChRs have been implicated in neuroprotection mechanisms against acute cell stress induced by excitotoxicity, namely overactivation of glutamate receptors^27^. The stimulation of α7 nAChRs by nicotine actually prevented glutamate-induced motor neuronal death^14^. Therefore, α7 nAChRs represent an important therapeutic target for ALS as well as for PD. Recently, a candidate ALS drug ropinirole (a dopamine D_2_ and D_3_ receptor agonist used as an antiparkinsonian drug) has been discovered in drug screenings using ALS patient-derived iPS cells, and is now being evaluated in clinical trials for effectiveness^28^. Although the mechanistic details are still unknown, there may be commonalities in the pathological mechanisms of PD and ALS. However, the effect of α7 nAChR agonists against mutant SOD1 aggregates in neurons remains unclear. A pathological hallmark of ALS is the presence of cytoplasmic inclusions or protein aggregates in affected motor neurons, suggesting that impairment of protein degradation may play a role in the disease pathology^29^. SOD1 aggregates are present in both sporadic and familial ALS^30,31^. In addition, we found that most wild-type SOD1 proteins assume misfolded conformations in cerebrospinal fluid of ALS patients regardless of *SOD1* mutation status^32^. Thus, removal of SOD1 aggregates may be a potential therapeutic approach for ALS treatment. Currently, more than 180 types of SOD1 pathogenic mutations have been identified in ALS patients^33^. Among those, the pathogenic SOD1^G85R^ mutation has been most frequently studied^34–37^.

As a strategy to remove mutant SOD1 aggregates, activation of autophagy, a prominent protein degradation pathway, has been reported to be effective in previous studies^38^. Autophagy is caused by the binding of lysosomes to autophagosomes which are formed by surrounding components to be degraded. Because previous studies have reported that activation of α7 nAChR induces autophagy, we are focusing on autophagy to elucidate the mechanism of the protective effect by activation of α7 nAChR in the present study^39,40^.

In the present study, we examined whether α7 nAChR activation exhibited neuroprotective effects against SOD1^G85R^-induced neurotoxicity in a cellular model of ALS.

## Results

### The α7 nAChR agonist reduces intracellular SOD1^G85R^ aggregates and prevents neurotoxicity of SOD1^G85R^

**Figure 1:**
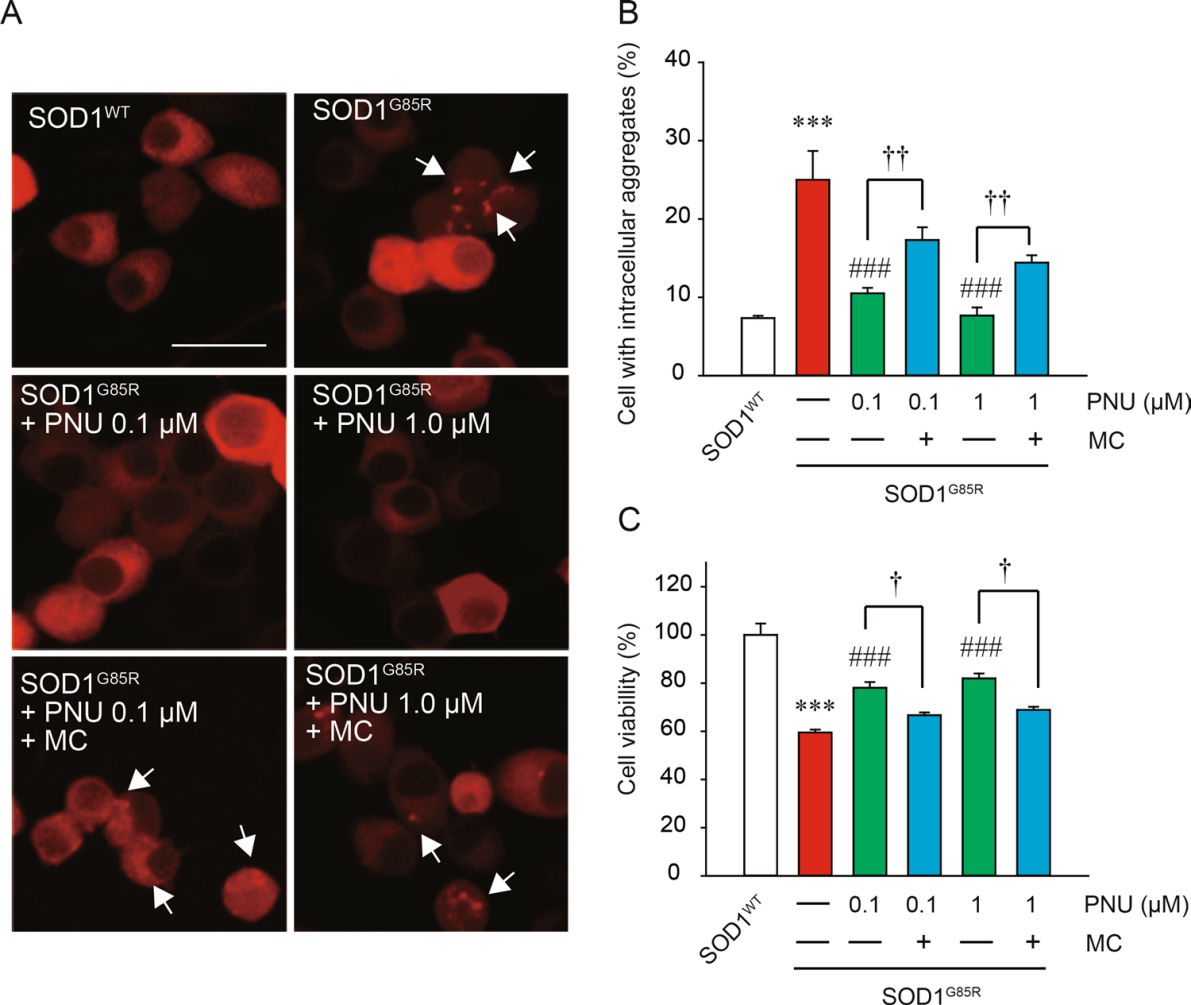
PNU282987 prevented SOD1^G85R^-induced neurotoxicity. At 24 h after transfection of each vector into N2a cells, the cells were treated with 0.1 or 1 µM PNU298987 for 24 h in the presence or absence of 20 µM methyllycaconitine (MC) pretreated before 30 min. (**A**) Representative fluorescent microscopy images. Scale bar: 10 µm. (**B**) Quantified data of intracellular SOD1 aggregates. (**C**) The cell viability was measured by MTT assay. Data is expressed as mean ± SEM from three independent experiments. Significance: ***p < 0.001 vs. SOD1^WT^; ^###^*p* < 0.001 vs. SOD1^G85R^; ^†^*p* < 0.05, ^††^*p* < 0.01 vs SOD1^G85R^ with PNU282987 by One-way ANOVA.

Previously, intracellular aggregate formation was confirmed in mCherry-fused SOD1^G85R^ (hereafter SOD1^G85R^)-transfected N2a cells, which contributed to cell death via the formation of triton X-100-insoluble aggregates^36–38,41^. To investigate the effect of α7 nAChR activation on SOD1^G85R^ aggregate formation, we evaluated the number of intracellular aggregates with an Imaging Cytometer after treatment with PNU282987, a selective nAChR agonist^39,42^ (Fig. 1). SOD1^G85R^ formed intracellular aggregates in approximately 25% of transfected N2a cells. PNU282987 treatment significantly reduced the percentage of cells with intracellular SOD1^G85R^ aggregates (Fig. 1A,B). This reduction in aggregate formation was markedly blocked by methyllycaconitine (MC), an α7 nAChR selective antagonist^43,44^ (Fig. 1A,B). In order to examine the effect of PNU282987 on SOD1^G85R^-mediated neurotoxicity, we performed a MTT assay in differentiated N2a cells (Fig. 1C). Although transfection of SOD1^WT^ did not affect cell survival, transfection of SOD1^G85R^ severely induced cell death. PNU282987 prevented SOD1^G85R^-induced cell death; in addition, MC significantly inhibited the PNU282987-induced neuroprotection (Fig. 1C). Besides, there was no effect of the treatment of MC alone on survival and aggregation (Supplemental Fig. S1). These results suggest that α7 nAChR activation exerts significant neuroprotective effects against SOD1^G85R^-induced toxicity via the reduction of intracellular protein aggregates. In addition, the expression level of the gene for α7 nAChR was not different in cells transfected with SOD1WT and G85R, which was confirmed by qRT-PCR (Supplemental Fig. S2).

### The α7 nAChR agonist exerts neuroprotective effects against SOD1^G85R^ aggregation via activation of autophagy

**Figure 2:**
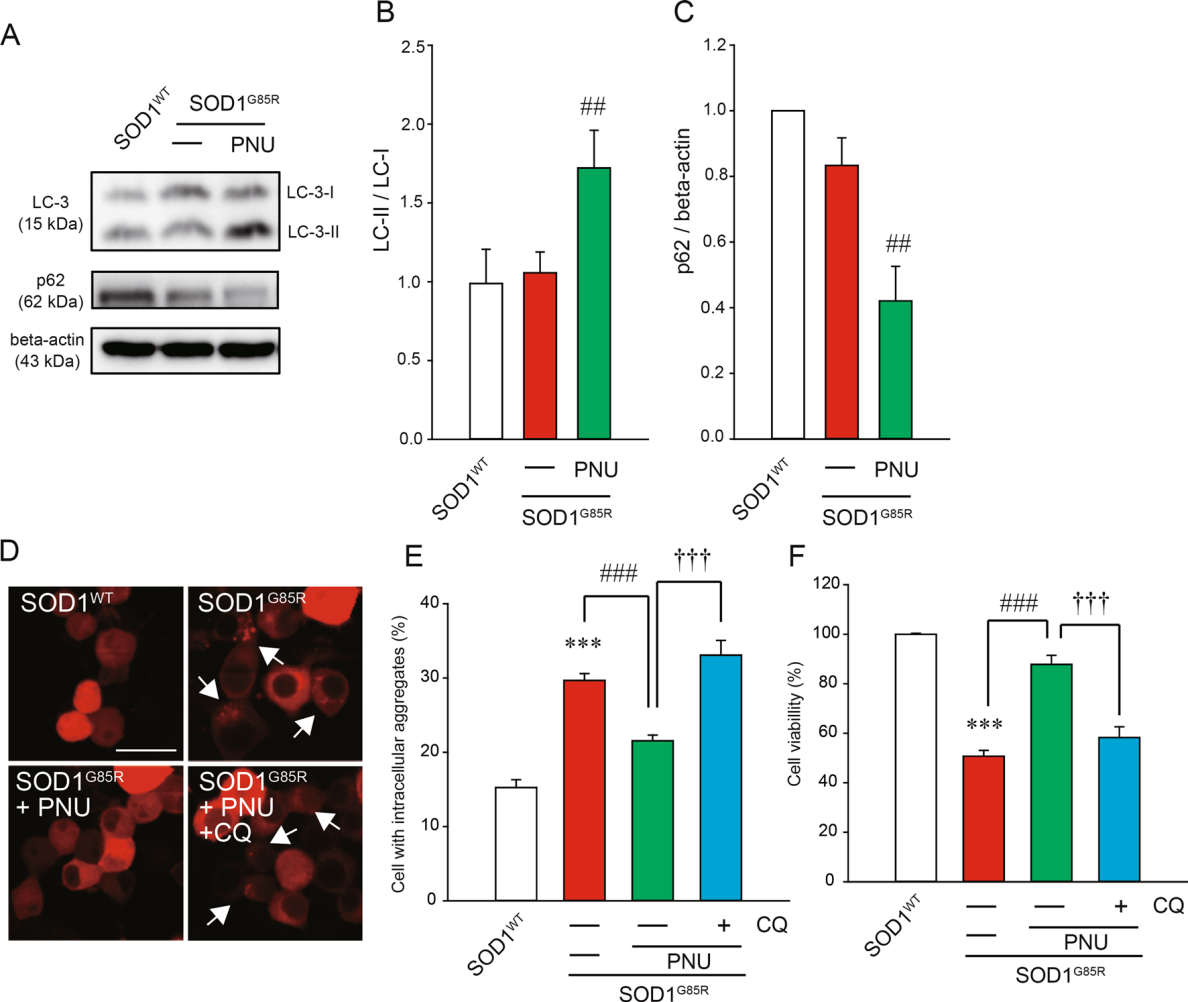
PNU282987 exerted the activation of autophagy. (**A**–**C**) After 24 h of transfection, N2a cells were transfected with SOD1^G85R^, and then incubated with 1 µM PNU282987. The lysates were analyzed by immunoblotting with anti-LC-3 (**A**,**B**) and p62 (**A**,**C**) and anti-β-actin antibodies. Levels normalized to the expression of β-actin and quantified based on the band density of SOD1^WT^. (**D**–**F**) N2a cells expressing mCherry-SOD1^G85R^ were treated with 1 µM PNU282987 (PNU) in the presence or absence of 20 nM chloroquine (CQ) pretreated before 30 min. (**D**) Representative fluorescent microscopy images. Scale bar: 10 µm. (**E**) Quantified data of intracellular SOD1 aggregates. (**F**) The cell viability was measured by MTT assay. Data is expressed as mean ± SEM from three independent experiments. Significance: ^###^*p* < 0.001 vs. SOD1^G85R^ by One-way ANOVA.

To analyze the mechanism of the reduction of intracellular aggregates upon α7 nAChR activation, we focused on autophagy, a prominent protein degradation pathway. The increase of LC3-II is a marker for the activation of autophagy because LC3-II converted from LC3-I is localized in the membrane of autophagosomes which is essential for autophagy and is proportional to the number of autophagosomes^45^. In addition, the decrease of p62 is also a marker of autophagy activation because p62 promotes degradation by autophagy and is itself degraded by autophagy^46^. Western blot analysis showed that PNU282987 treatment increased the formation of LC3-II in N2a cells (Fig. 2A,B). In addition, similar results were confirmed for calculations by taking the ratio of beta-actin (Supplemental Fig. S3). PNU282987 treatment also significantly decreased p62 protein levels (Fig. 2A,C). In addition, chloroquine (CQ), an inhibitor of autophagy, prevented the reduction of cytoplasmic aggregation of SOD1^G85R^ induced by PNU282987 (Fig. 2D,E). To further investigate whether the neuroprotective effects of PNU282987 were associated with autophagy, we performed a MTT assay in the presence of CQ. The protective effect of PNU282987 was significantly inhibited by CQ treatment (Fig. 2F). Besides, there was no effect of the treatment of CQ alone on survival and aggregation (Supplemental Fig. S1). These data suggest that PNU282987 treatment reduces the quantity of subcellular aggregates via the upregulation of autophagy, which prevented SOD1^G85R^-associated neurotoxicity.

### α7 nAChR activation promotes autophagy via Ca^2+^ influx and the AMP-activated protein kinase (AMPK)-mammalian target of rapamycin (mTOR) signaling pathway

**Figure 3:**
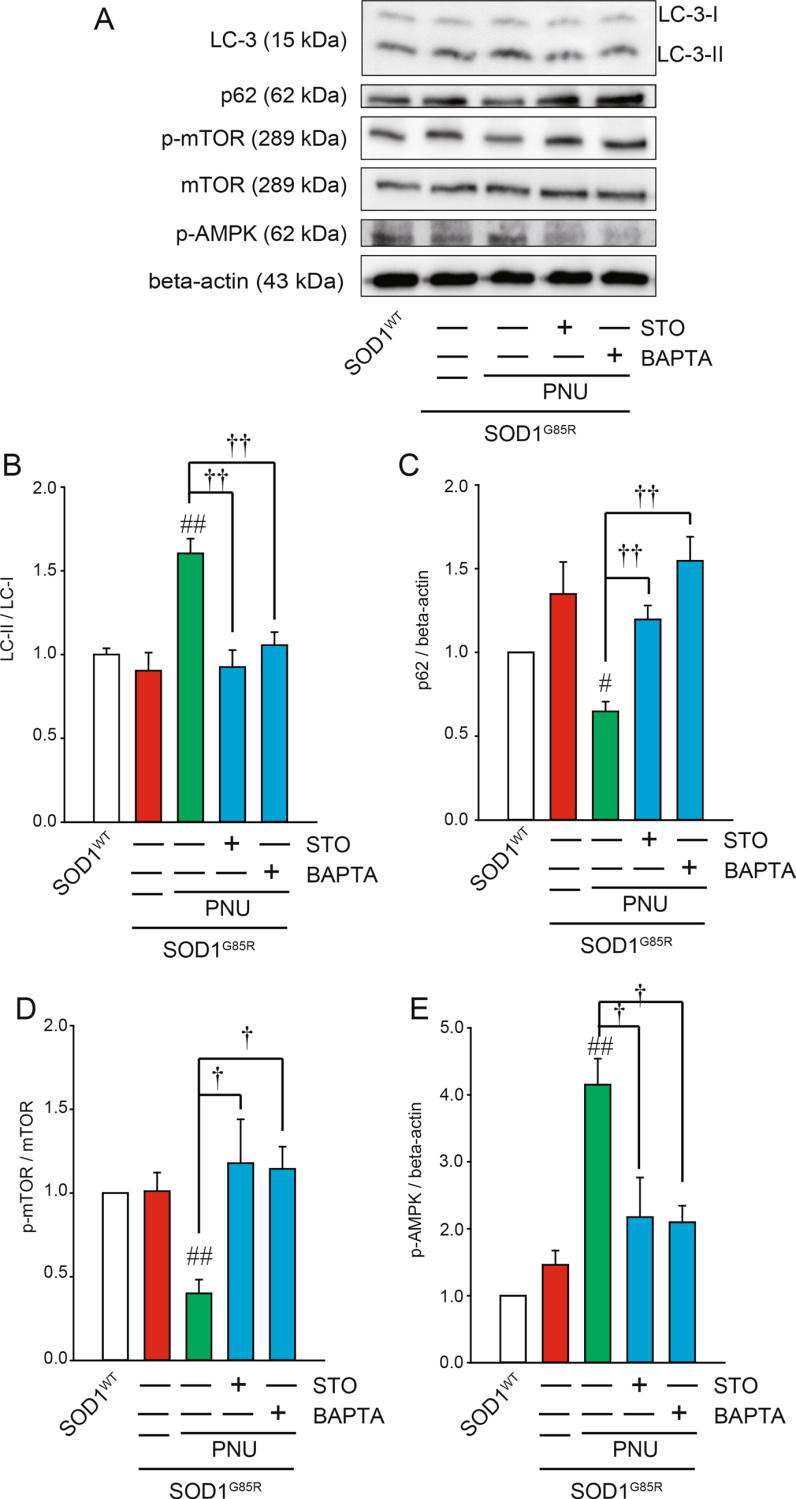
PNU282987-induced Ca^2+^ influx contributes to autophagy. Immunoblotting analysis of autophagy regulators with a Ca^2+^ chelator and CaMKK-specific inhibitor. N2a cells expressing SOD1^G85R^ were treated with 1 µM PNU282987 (PNU) in the presence or absence of 1 µM STO609 (STO) or 1 µM BAPTA-AM (BAPTA) pretreated before 30 min. (**A**) The lysates were analyzed by immunoblotting with antibodies for LC-3, p62, phosphorylated mTOR (p-mTOR), mTOR, phosphorylated AMPK (p-AMPK), β-actin. (**B**–**E**) Relative levels normalized by the expression of LC3-I or mTOR or β-actin were quantified, based on the density of SOD1^WT^. Data is expressed as mean ± SEM from three independent experiments. Significance: ^#^*p* < 0.05, ^##^*p* < 0.01 vs. SOD1^G85R^; ^†^p < 0.05, ^††^p < 0.01 vs SOD1^G85R^ with PNU282987 by One-way ANOVA.

α7 nAChR activation increases intracellular Ca^2+^ influx through the α7 receptor channel. It is well-established that Ca^2+^ influx is a regulator of autophagy^47^. To elucidate the pathways involved in elevated Ca^2+^ influx after α7 nAChR activation, we used the cell-permeable cytosolic Ca^2+^ chelator 1,2-bis (2-aminophenoxy) ethane-N,N,N0,N0-tetraacetic acid tetrakis (acetoxymethyl ester) (BAPTA-AM)^48,49^. Pre-treatment with BAPTA-AM significantly inhibited the formation of LC3-II and the reduction in p62 protein induced by PNU282987 (Fig. 3A–C). CaMKKβ has been proposed as a potential target of cytosolic Ca^2+^ signaling^50^, although direct evidence remains elusive. Similar to the effects of BAPTA-AM, the formation of LC3-II and the reduction in p62 induced by PNU282987 were significantly inhibited by STO609, a CaMKKβ-specific inhibitor^51,52^ (Fig. 3A–C).

The AMP-activated protein kinase (AMPK)–mammalian target of rapamycin (mTOR) signaling pathway is downstream of Ca^2+^ signaling and plays an important role in the regulation of autophagy in response to different stress conditions^53^. To further identify the signal transduction pathway mediated by PNU282987, we investigated the AMPK–mTOR pathway. PNU282987 significantly increased AMPK phosphorylation and significantly inhibited mTOR phosphorylation (Fig. 3A,D,E). In addition, BAPTA-AM and STO609 pre-treatment significantly decreased PNU282987-dependent AMPK phosphorylation and restored mTOR phosphorylation (Fig. 3A,D,E), suggesting activation of the AMPK pathway and inhibition of the mTOR pathway occurs via Ca^2+^ influx following α7 nAChR activation. These results suggest that α7 nAChR activation potentially induces autophagy via Ca^2+^ influx and signaling through the AMPK and mTOR pathways.

### α7 nAChR agonist promotes lysosomal activation via nuclear translocation of transcription factor EB (TFEB)

**Figure 4:**
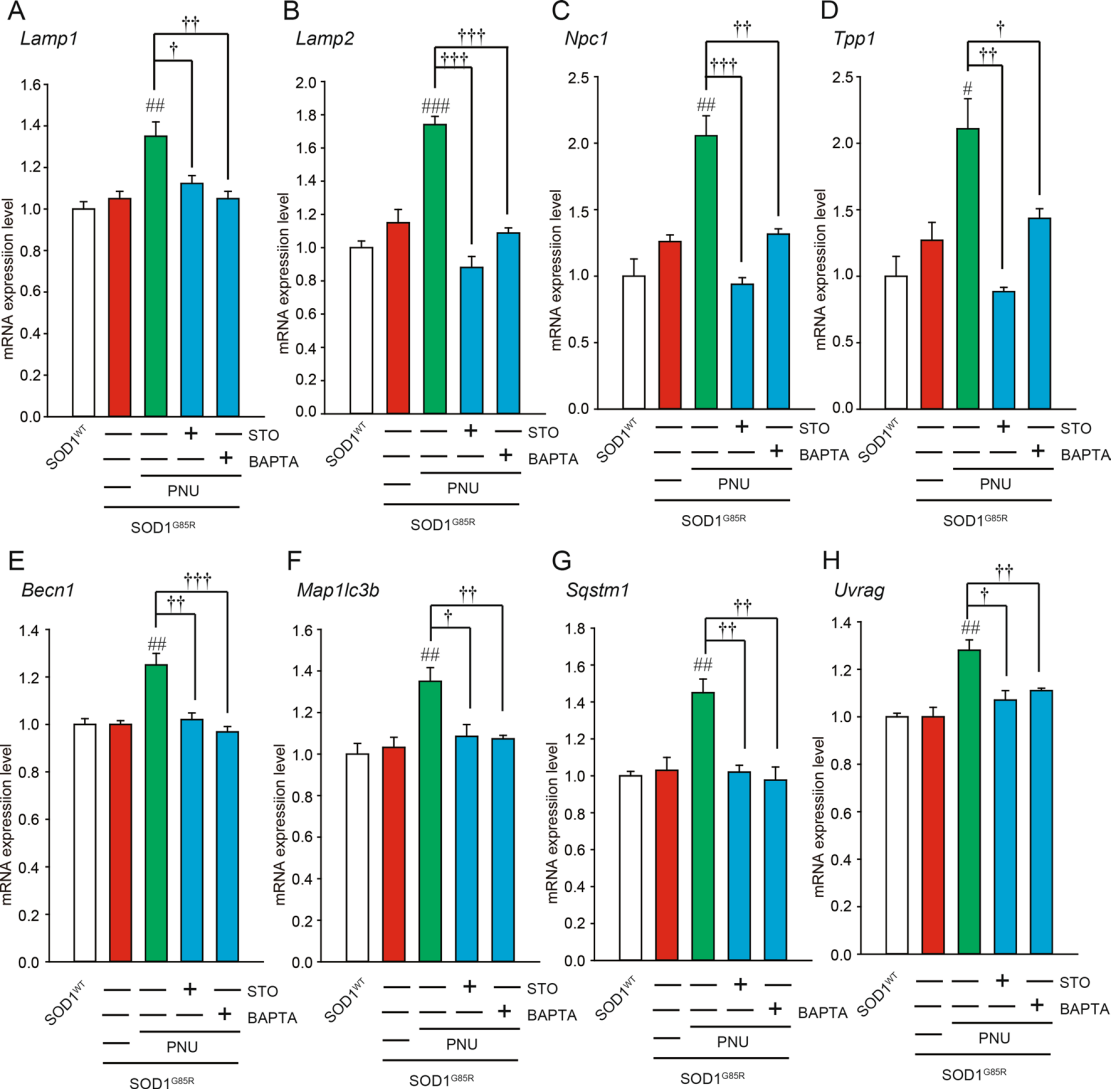
Effects of PNU282987 on transcription factor EB (TFEB) related mRNA expression. (**A**–**H**) N2a cells expressing mCherry-SOD1^G85R^ were treated with 1 µM PNU282987 (PNU) in the presence or absence of 1 µM STO609 (STO) and 1 µM BAPTA-AM (BAPTA) pretreated before 30 min. At 24 h after treatment with PNU, mRNA expressions of *Lamp1* (**A**)*, Lamp2* (**B**)*, Npc1* (**C**)*, Tpp1* (**D**)*, Becn1* (**E**)*, Map1lc3b* (**F**)*, Sqstm1* (**G**)*, Uvrag* (**H**) were analyzed using the SYBR Green-based RT-qPCR assay. The expression levels of mRNA were normalized to the expression levels of β-actin mRNA. Significance: ^#^*p* < 0.05, ^##^*p* < 0.01, ^###^*p* < 0.01 vs. SOD1^G85R^; ^†^*p* < 0.05, ^††^*p* < 0.01, ^†††^*p* < 0.01 vs SOD1^G85R^ with PNU282987 by One-way ANOVA.

AMPK signaling activates transcription factor EB (TFEB), which is a potential key transcription in the induction of autophagy upon its dephosphorylation and nuclear translocation^54^. TFEB can enter the nucleus and bind to the E-box of the CLEAR element, which regulates the transcription of genes associated with the biogenesis of lysosomes and autophagosomes^55^. We first analyzed mRNA levels of lysosomes and autophagy-related genes whose transcription could be regulated by TFEB using qRT-PCR. The mRNA levels of lysosomal-related genes (such as *Lamp1*, *Lamp2*, *Npc1*, and *Tpp1*) and autophagy-related genes (such as *Becn1, Map1lc3b, Sqstm1,* and *Uvrag*) were significantly up-regulated in response to PNU282987 treatment (Fig. 4). Upregulation of these mRNAs was markedly blocked by pre-treatment with BAPTA-AM and STO609 (Fig. 4).

**Figure 5:**
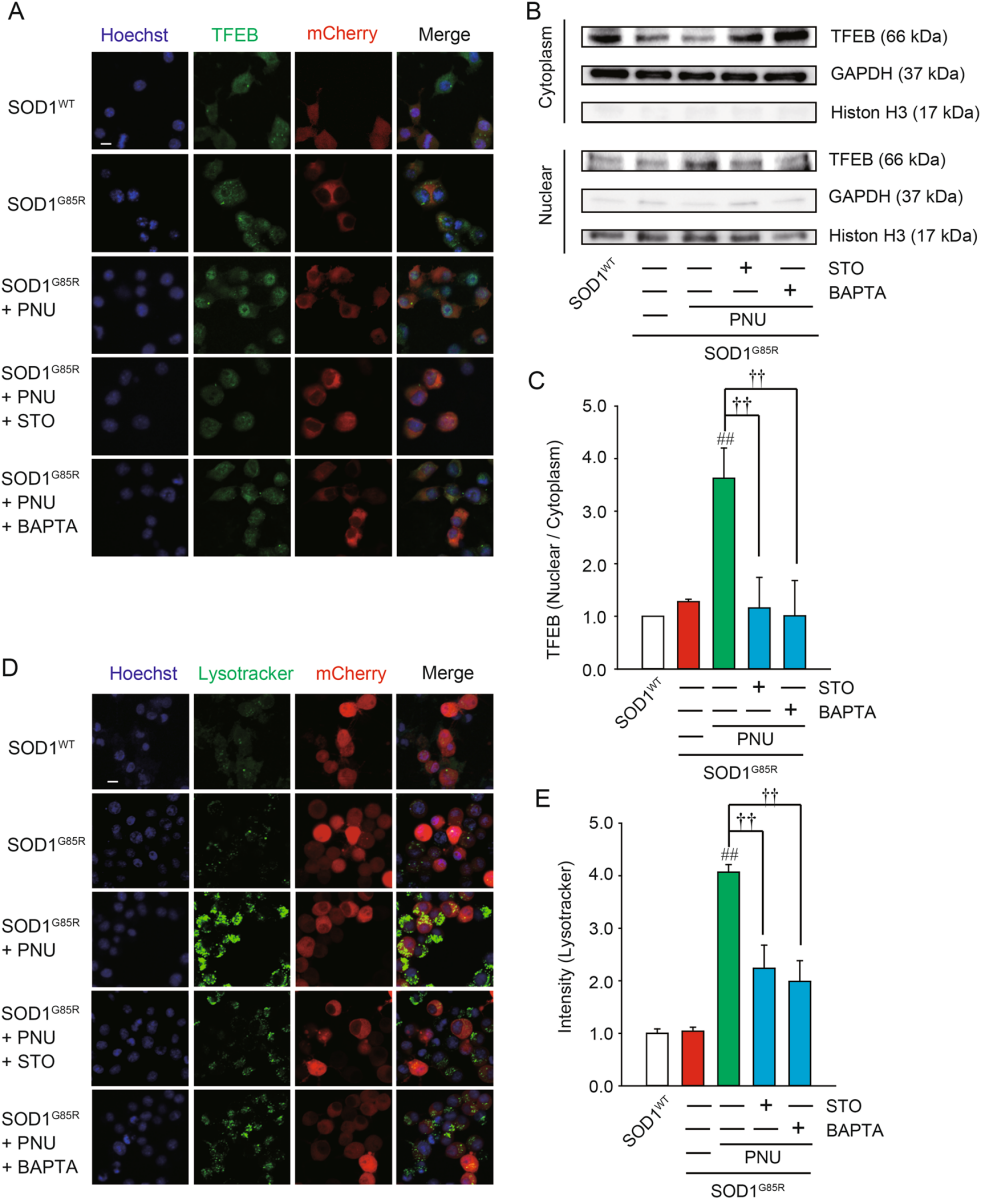
PNU282987 enhances TFEB activity. N2a cells expressing SOD1^G85R^ were treated with 1 µM PNU282987 (PNU) in the presence or absence of 1 µM STO609 (STO) or 1 µM BAPTA-AM (BAPTA) pretreated before 30 min. (**A**) Representative fluorescent microscopy images of TFEB. Scale bar: 10 µm. (**B**) After the fractionation using NE-PER Nuclear and Cytoplasmic Extraction Reagents, the lysates were analyzed by immunoblotting with antibodies for TFEB, GAPDH, and Histone H3. (**C**) The ratio of nuclear to cytosolic TFEB was calculated and analyzed. (**D**) Representative fluorescent microscopy images of lysotracker. Scale bar: 10 µm. (**E**) Quantified data of lysotracker intensity. Significance: ^##^*p* < 0.01 vs. SOD1^G85R^; ^††^p < 0.01 vs. SOD1^G85R^ with PNU282987 by One-way ANOVA.

Next, we examined the distribution of TFEB in the SOD1^G85R^-transfected N2a cells after PNU282987 treatment using immunofluorescence (Fig. 5A). TFEB levels in the nucleus were increased following PNU282987 treatment. Meanwhile, TFEB levels in the cytoplasm were decreased by PNU282987 (Fig. 5A). Western blot also demonstrated that PNU282987 treatment decreased and increased TFEB levels in cytoplasmic and nuclear fractions, respectively (Fig. 5B,C). In addition, nuclear translocation of TFEB induced by PNU282987 was significantly inhibited following pre-treatment with BAPTA-AM and STO609 (Fig. 5B,C). We next determined whether PNU282987 enhances lysosomal activation using immunofluorescence (Fig. 5D,E). PNU282987 promoted lysosomal activation as quantified by fluorescence intensity in LysoTracker-stained cells. The effect of PNU282987 on lysosomal activation was inhibited by pre-treatment with BAPTA-AM and STO609 (Fig. 5D,E). Together, these data suggest that α7 nAChRs activation promotes lysosomal activation via the nuclear transposition of TFEB.

## Discussion

The goal of the present study was to determine whether activation of α7 nAChR had a neuroprotective effect against SOD1^G85R^ aggregate-induced toxicity in a cellular model of ALS. We demonstrated that PNU282987, an α7 nAChR agonist, induces significant neuroprotective effects via reduction of SOD1^G85R^ intracellular aggregates. This reduction strongly correlated with the activation of autophagy via the AMPK–mTOR signaling pathway. Furthermore, PNU282987 increased lysosomal activation by promoting TFEB translocation into the nucleus. These findings identify α7 nAChR as a novel neuroprotective target against the SOD1^G85R^ aggregate-mediated neurotoxicity. Besides, because of the transient expression of the SOD1 system in this study, there may be a slight change in the gene transfer efficiency. The difference may affect the change in aggregation rate. However, we can always see an increase in aggregation rate in transfected cells with SOD1^G85R^ compared to SOD1^WT^, therefore, we think that it does not affect the experimental results. In the future, we would like to examine them in a more pathological model using iPS cells rather than this model of transient expression.

Autophagy dysfunction has been implicated in various neurodegenerative diseases, including ALS^56,57^. The notion that autophagy dysfunction contributes to ALS pathogenesis is strongly supported by the identification of numerous genes associated with familial ALS also involved in regulation of autophagy, including *SQSTM1*^58^, *OPTN*^4^, *TBK1*^59^, and *VCP*^60^. In addition, previous reports also showed that TFEB downregulation induced autophagy defect in spinal motor neurons of patients with SOD1^G93A^ mutations although not confirmed in the mutant SOD1^G85A^ model in this study^61^. This difference in results may be due to differences in cell type. Therefore, further research is needed on other models. Besides, the presence of intracellular, insoluble inclusions composed of misfolded proteins is a hallmark of ALS pathology^29^. Therefore, removal of SOD1 aggregates may represent one potential therapeutic approach for ALS treatment.

There are two major pathways for cellular protein degradation: the ubiquitin proteasome system (UPS), and autophagy. Autophagy has been shown to degrade both soluble and aggregated protein substrates that are too large to pass through the pore of the proteasome, such as the toxic SOD1 protein aggregates, while UPS primarily degrades soluble SOD1, suggesting that autophagy regulation is critical for improving ALS pathology^57,62^.

Treatment of mutant SOD1 transgenic mice with trehalose resulted in increased life span, improved neuronal survival, reduced astrogliosis, and delayed disease onset via activation of autophagy^63^. Similarly, carbamazepine treatment activated autophagy via the AMPK-ULK1 signaling pathway and promoted the clearance of mutant SOD1 aggregates. Carbamazepine treatment also delayed disease onset and extended life span of SOD1^G93A^ mice^64^. In our previous studies, autophagy induction has demonstrated beneficial effects in cells harboring pathogenic SOD1 mutations^36,37,65^. In addition to pharmacological studies, genetic ablation of XBP-1 (X-box-binding protein) in motor neurons of SOD1^G85R^ mice enhanced the clearance of mutant SOD1 aggregates and increased survival via activation of autophagy^66^. Moreover, bosutinib, which boosts autophagy, can improve the survival of iPS cells-derived motor neurons from patients with familial ALS caused by mutations in SOD1^67^.

Conversely, abnormalities (activations) in autophagy have been observed in numerous neurodegenerative diseases, including ALS^68^. Pharmacological and genetic modulation of autophagy may result in diverse and even detrimental outcomes to the survival of ALS models; interventions targeting genes including mSOD1, FUS and TDP-43^57,62^ have shown that it may be necessary to jointly consider the specific effects of each individual mutation, pathology, and possibly other context-dependent influences. These results suggest the need for developing autophagy inducers with higher specificity and lower cytotoxicity based on ALS pathology^62^. In the present study, PNU282987 exerted neuroprotective effects against SOD1^G85R^-induced toxicity via autophagy activation. Although it is necessary to explore this finding further with iPS cells and animal models, among candidates of autophagy inducers, α7 nAChR may be a promising candidate.

Our study indicates that PNU282987 decreased mTOR phosphorylation and increased AMPK phosphorylation, and subsequently induced autophagy. The AMPK–mTOR signaling pathway is a downstream target of Ca^2+^ signaling and plays an important role in the regulation of autophagy in response to different stresses^53^. In support of this, we showed that AMPK and mTOR phosphorylation were significantly affected by pre-treatment with BAPTA-AM and STO609, indicating activation of the AMPK pathway and inhibition of the mTOR pathway via Ca^2+^ influx following α7 nAChR activation. In addition, AMPK phosphorylation activates TFEB, which is a potential key transcription for autophagy induction upon its dephosphorylation and nuclear translocation^54^. It has been reported that under stress conditions or upon loss of function, TDP43 can regulate the nuclear translocation of TFEB in order to promote the transcription of autophagic genes. This indicates that TFEB may play a role in potential strategies for ALS treatment. In the present study, PNU282987 significantly increased the mRNA levels of *Lamp1*, *Lamp2*, *Npc1*, *Tpp1, Becn1, Map1lc3b, Sqstm1, and Uvrag* the transcription of which could be regulated by TFEB. In addition, PNU282987 significantly increased the TFEB translocation into the nucleus and promoted lysosomal activation. Previously, trehalose, an enhancer of mTOR-independent autophagy, was shown to delay ALS onset and reduce motor neuron loss in SOD1^G93A^ mice^69^. In contrast, mTOR-dependent activation of autophagy resulted in loss of motor neurons and reduced survival in the same ALS mouse model, which may be due to other physiological functions of mTOR inhibition^70^. The neuroprotective effects of α7 nAChR activation may be due to pleiotropic effects including other signaling pathways such as FGFR1 and NF-κB, it will be necessary to conduct further studies^71–73^. In addition, further research is needed on other possibilities for other receptors, such as α4β2 nAChR, the other major nAChR.

ALS is a multifactorial disease encompassing a network of cellular pathways^74^. Drugs with pleiotropic effects may be practically more effective than drugs with a single effect for patients with ALS. As α7 nAChR activation has various neuroprotective effects including autophagy activation, α7 nAChR activation may possess novel therapeutic potential for ALS. Recently, the usefulness of patient-derived iPS cells as a model for ALS has been demonstrated^28,67^. Therefore, further research is required at a level closer to the pathological conditions, such as using iPS cells.

## Material and methods

### Plasmid, cell culture, and transfection

Expression plasmids (pmCherry-N1, Clontech Laboratories Inc., CA, USA) harboring human SOD1 (Wild-type (SOD1^WT^) or mutant (SOD1^G85R^)) were prepared as previously reported^36,37^. Briefly, N2a cells (Mouse Albino neuroblastoma, ECACC, UK) were maintained in Dulbecco’s modified Eagle medium (DMEM, Wako Pure Chemical Industries, Ltd.) containing 10% (v/v) fetal bovine serum (FBS; Thermo Fisher Inc.) under a humidified atmosphere of 5% CO_2_ at 37 °C. The cells were passaged by trypsinization every 3–4 days. The transient plasmid expression in N2a cells was accomplished with Lipofectamine 2000 according to the manufacturer’s protocol (Thermo Fisher Scientific Inc.).

### Thiazolyl blue tetrazolium bromide (MTT) assay

N2a cells were seeded at 2.0 × 10^5^ cells/ml in 96-well plates in DMEM containing 10% FBS. Following 24 h of plasmid transfection, the cells were differentiated for 48 h in low glucose (1.0 g/l) DMEM supplemented with 2% FBS and 2 mM *N*,*N*-dibutyladenosine 3′,5′-phosphoric acid (dbcAMP; Nacalai Tesque Inc.) with 0.1 or 1 µM PNU282987 (Wako Pure Chemical Industries Ltd.) in the presence or absence of 20 µM MC (Cayman Chemical Ltd.) or 20 nM CQ (Tokyo Chemical Industry Ltd.) pretreated before 30 min. The number of live cells was estimated by Cell Counting Kit-8, following the manufacturer’s instructions (Wako Pure Chemical Industries Ltd.). Briefly, the reagent was added into the wells and the plate was incubated at 37 °C for 4 h. Cell viability was calculated through the detection of the optical density of formazan at 450 nm using GloMax (Promega). A 600 nm wavelength was used as reference.

### Aggregation rate analysis

We performed aggregation assay based on previous studies^36,37^. Briefly, after 24 h of mCherry-fused SOD1^WT^ or SOD1^G85R^ vector transfection, the cells were treated with PNU282987 for 24 h in the presence or absence of 20 µM MC or 20 nM CQ pretreated before 30 min. Subsequently, the cells were washed twice with PBS for 5 min and fixed with 4% paraformaldehyde for 15 min. Fluorescent microscopy images were acquired with a confocal fluorescence microscope (LSM700, Carl Zeiss). For counting the number of aggregates, we used IN Cell Investigator 2200 (GE Healthcare). In each experiment, at least 3000 cells were counted.

### Immunoblotting

At 24 h after transfection of each vector into N2a cells, the cells were treated with 1 µM PNU298987 for 24 h in the presence or absence 1 µM STO609 or 1 µM BAPTA-AM pretreated before 30 min. After treatment, the cells were lysed with TNE lysis buffer (50 mM Tris–HCl (pH. 7.4), 150 mM NaCl, 1 mM ethylenediamineteraacetic acid, protease inhibitor cocktail) containing 1% Triton X and then were centrifuged at 15,000×*g* at 4 °C for 5 min. The supernatant protein sample was collected. Protein concentrations were quantified using a BCA protein assay kit (Thermo Fisher Scientific Inc.) with bovine serum albumin (BSA) as a standard. Lysates were mixed with sample buffer containing 10% 2-mercaptoethanol, and subjected to 10% SDS–polyacrylamide gel electrophoresis (SDS-PAGE). SDS-PAGE was performed under constant voltage of 200 V at room temperature for 40 min. The separated proteins in polyacrylamide gel were transferred to a PVDF membrane in transfer buffer (0.3% Tris, 1.44% glycine, 20% methanol) under constant voltage of 100 V at 4 °C for 90 min. The membranes were incubated with 5% BSA (Wako) for 60 min, and then with following primary antibodies for overnight: mouse monoclonal antibody against β-actin (1: 2000, Santa Cruz Biotechnology) and rabbit polyclonal antibodies against LC-3 (1:1000, Cell Signaling), p-AMPK (1:1000, Cell Signaling Technology), p-mTOR (1:1000, Cell Signaling Technology), mTOR(1:1000, Cell Signaling Technology), p62 (1:1000, Cell Signaling Technology), TFEB (1:1000, Proteintech). After the primary antibody reaction, the membrane was incubated in the secondary antibody (goat anti-rabbit antibody conjugated with HRP (1:2500, Santa Cruz Biotechnology) or goat anti-mouse HRP antibody conjugated with HRP (1:2500, Santa Cruz Biotechnology)). The membrane was incubated in ECL prime (GE Healthcare, Buckinghamshire, UK) to generate the chemiluminescence from HRP antibodies. The chemiluminescence was detected by Fusion System (Vilber-Lourmat). The band density was measured using ImageJ.

### RNA preparation and qRT-PCR

Reverse transcription was performed using the ReverTra Ace qPCR RT Master Mix, in accordance with the manufacturer’s instructions (TOYOBO). qRT-PCR was performed using SYBR Green on a StepOne Real-Time PCR System, in accordance with the manufacturer’s instructions (Life Technologies). The sequences of gene-specific primer sets are shown in Table 1. The expression levels of mRNA were normalized to the expression levels of β-actin mRNA.

**Table 1:** Primer pairs used for qRT-PCR.

	Forward	Reverse
*Lamp1*	5′tcaaggtggacagtgacaggt3′	5′tgactcctcttcctgccaatga3′
*Lamp2*	5′tctccggttaaaggcgcaaag3′	5′tcatctcccattctgcataaaggc3′
*Npc1*	5′cgcaatcctgtgtttggtatgg3′	5′aagtcatagccgtcctttggg3′
*Tpp1*	5′cccatgttataaggtccccacatcc3′	5′ccaagtgcaggctaacagttcc3′
*Becn1*	5′gcggagagattggaccagga3′	5′tctccacactcttgagttcgtca3′
*Map1lc3b*	5′gtgcctgaccacgtgaacat3′	5′tctcactctcgtacacttcgga3′
*Sqstm1*	5′cagatgccagaatcggaaggg3′	5′ggactcaatcagccggggat3′
*Uvrag*	5′ctgtgtcctgctttgtggtga3′	5′tttcattctggttgcgggca3′
*Chrna7*	5′gcccttgatagcacagtacttcg3′	5′gatcctggtccacttaggcattt3′
*β-actin*	5′cgttgacatccgtaaagacc3′	5′gctaggagccagagcagtaa3′

*Lamp1* lysosome-associated membrane protein 1, *Lamp2* lysosome-associated membrane protein 2, *Npc1* NPC intracellular cholesterol transporter 1, *Tpp1* tripeptidyl peptidase 1, *Becn1* Beclin1, *Map1lc3b* microtubule associated protein 1 light chain 3 beta, *Sqstm1* Sequestosome 1, *Uvrag* UV radiation resistance associated, *Chrna7* cholinergic receptor nicotinic alpha 7.

### Immunostaining

At 24 h after transfection of each vector into N2a cells, the cells were treated with 1 µM PNU298987 in the presence or absence 1 µM STO609 or 1 µM BAPTA-AM pretreated before 30 min. At 24 h after the treatment, cells were fixed by 4% paraformaldehyde and then permeabilized with 0.1% Triton X-100 diluted in PBS. To block the reaction, the cells were treated with 2% goat serum for 60 min. The cells were incubated with rabbit polyclonal antibody against TFEB (1:200, Proteintech) at 4 °C for overnight. Subsequently, the cells were incubated with secondary antibody (goat anti-rabbit antibody Alexa 488) at room temperature for 1 h. In addition, nuclear staining was performed with Hoechst 33342 (Molecular Probes). Fluorescent microscopy images were acquired with a confocal fluorescence microscope (LSM700, Carl Zeiss).

### Lysosomal staining

N2a cells were seeded at 1.0 × 10^5^ cells/ml in CELLview cell culture dishes (Greiner Bio-one). At 24 h after transfection to N2a cells each vector, the cells were treated with 1 µM PNU298987 in the presence or absence 1 µM STO609 or 1 µM BAPTA-AM pretreated before 30 min. At 24 h after the treatment, lysosomes were stained with LysoTracker Green DND-26 (Thermo Fisher Scientific) and nucelei were stained with Hoechst 33342 (Thermo Fisher Scientific), in accordance with the manufacturer’s instructions (Thermo Fisher Scientific). Fluorescent microscopy images were acquired with a confocal fluorescence microscope (LSM700, Carl Zeiss). Image analysis was calculated the fluorescence intensities in each image using ImageJ (thresholding and then brightness measurement) and took the ratio by the number of cells stained with Hoechst.

### Cell fractions preparation

At 24 h after transfection of each vector into N2a cells, the cells were treated with 1 µM PNU298987 in the presence or absence 1 µM STO609 or 1 µM BAPTA-AM pretreated before 30 min. At 24 h after the treatment, 1.0 × 10^6^ cells were harvested with trypsin–EDTA and nuclear extraction was performed using NE-PER Nuclear and Cytoplasmic Extraction Reagents, in accordance with the manufacturer’s instructions (Thermo Fisher Scientific). To assess the purity of the fractionation, the cytoplasmic and nuclear fractions were confirmed by immunoblotting using anti-GAPDH (1:1000, MBL Ltd.) as a cytoplasmic marker and anti-Histone H3 (1:1000, Cell Signaling Technology Ltd.) as a nuclear marker.

### Statistical analysis

Data are given as the mean ± standard error of the mean (SEM). Significance was determined using the analysis of variance. Further statistical analysis for post hoc comparisons was performed using the Bonferroni/Dunn test (StatView, Abacus). p-values of less than 0.05 were considered to be statistically significant.

## Supplementary Information

Supplementary Information.

## Data Availability

The datasets used and/or analysed during the current study are available from the corresponding author on reasonable request.
