# Supplementary material for: The neuroprotective effects of activated α7 nicotinic acetylcholine receptor against mutant copper–zinc superoxide dismutase 1-mediated toxicity
Source: Sci Rep. 2020 Dec 17;10:22157. doi: 10.1038/s41598-020-79189-y (PMC7746719; doi:10.1038/s41598-020-79189-y)
Supplement: Supplementary file 1 — Supplementary Information. [file 41598_2020_79189_MOESM1_ESM.docx]

**Revised Sup: f317aa32-0c32-4cb8-89c7-0241c4feacd1**

**Supplementary Information**

**The neuroprotective effects of activated α7 nicotinic acetylcholine receptor against mutant copper-zinc superoxide dismutase 1-mediated toxicity**

Taisei Ito^1^*, Masatoshi Inden^1^*, Tomoyuki Ueda^1^, Yuta Asaka^1^, Hisaka Kurita^1^ & Isao Hozumi^1^

^1^Laboratory of Medical Therapeutics and Molecular Therapeutics, Gifu Pharmaceutical University

*These authors contributed equally to this work.

***Corresponding author**: **Isao Hozumi, M.D., Ph.D.**

Laboratory of Medical Therapeutics and Molecular Therapeutics,

Gifu Pharmaceutical Univ., 1-25-4 Daigaku-nishi, 1-1-1 Gifu 501-1196, Japan

TEL/FAX: +81(Japan)-58-230-8121

E-mail: [hozumi@gifu-pu.ac.jp](mailto:hozumi@gifu-pu.ac.jp)

**(a)**


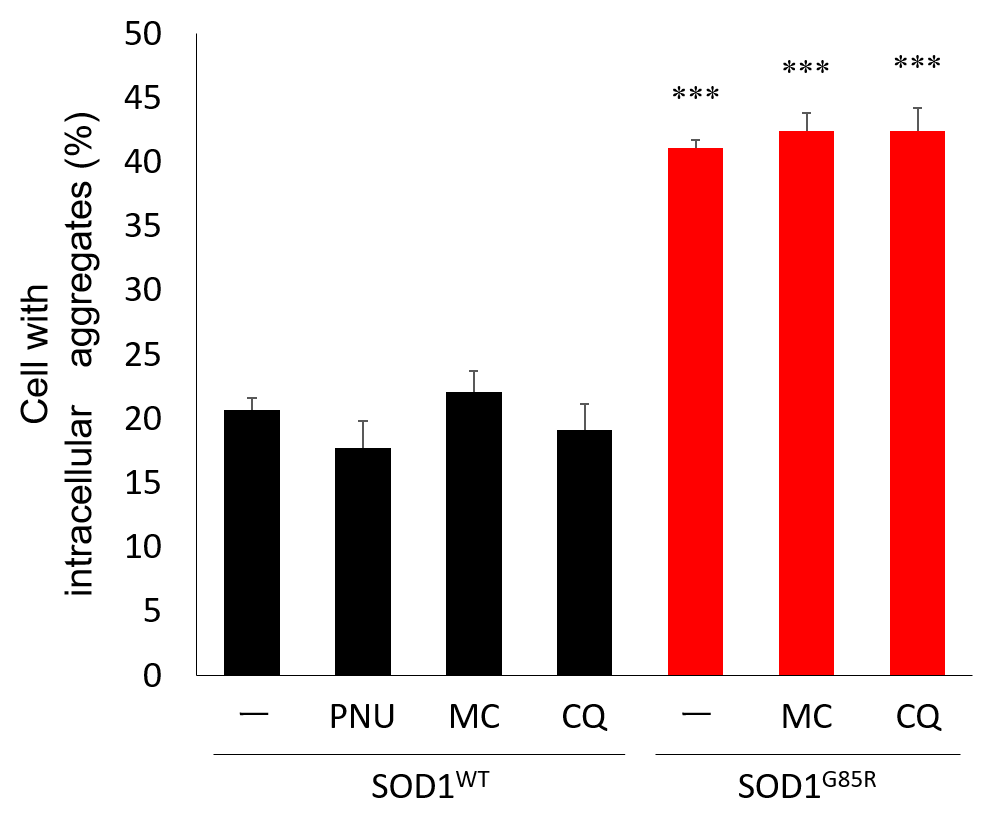


**(b)**


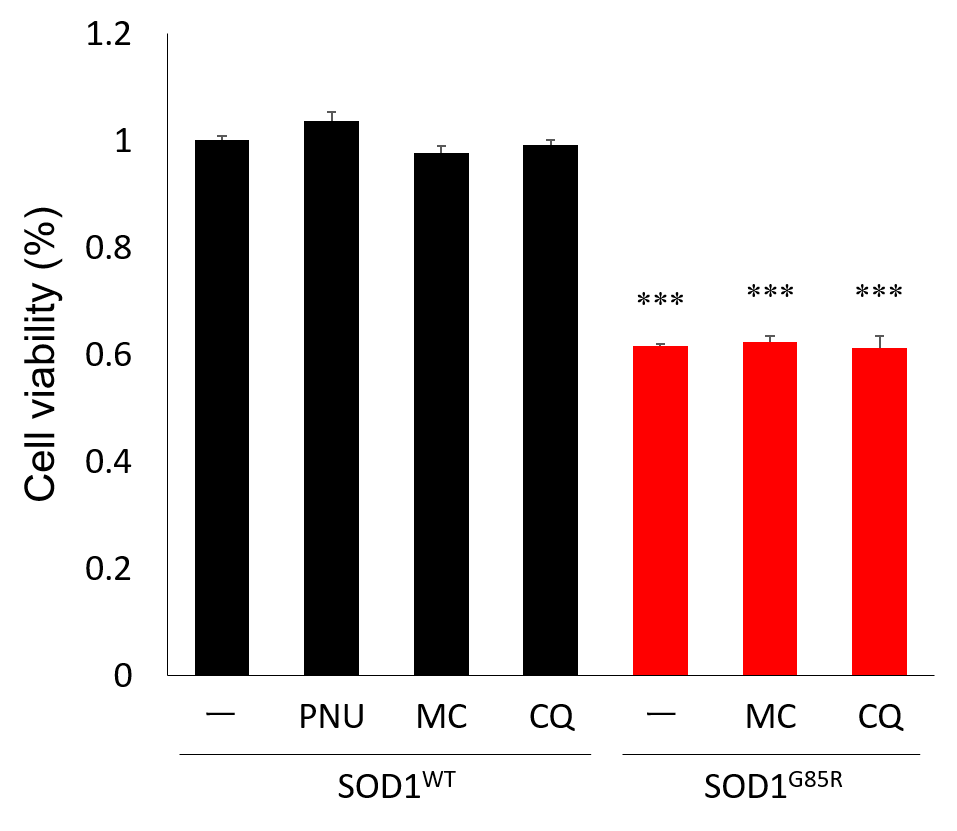


**Supplementary Figure 1.** After 24 h of transfection, N2a cells expressing mCherry-SOD1WT, SOD1G85R were treated with 1 µM PNU282987 (PNU), 20 µM methyllycaconitine (MC), 20 nM chloroquine (CQ). (a) Quantified data of intracellular SOD1 aggregates. (b) The cell viability was measured by MTT assay. Data is expressed as mean ± SEM from three independent experiments. Significance: *** p < 0.001 vs. SOD1WT (-).


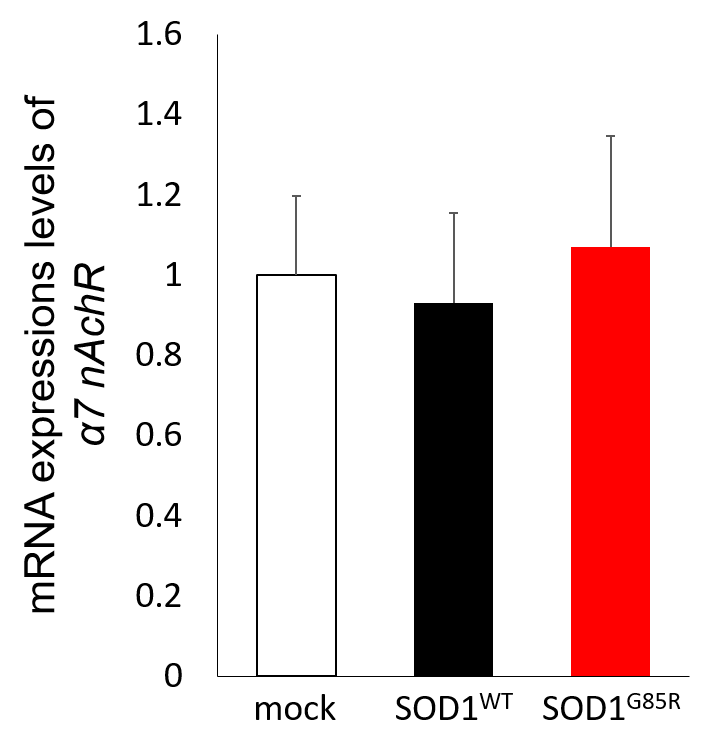


**Supplementary Figure 2.** N2a cells transfected SOD1WT, SOD1G85R. At 24 h after transfection, mRNA expressions of α7 nAChR were analyzed using the SYBR Green-based RT-qPCR assay. The expression levels of mRNA were normalized to the expression levels of β-actin mRNA. Data is expressed as mean ± SEM from three independent experiments. Significance: Nonsignificant vs. SOD1WT.

**(a)**


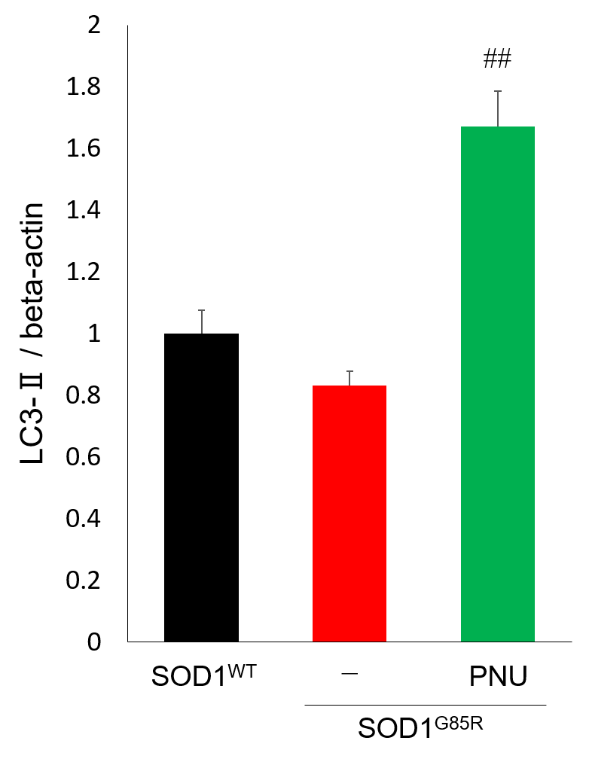


**(b)**


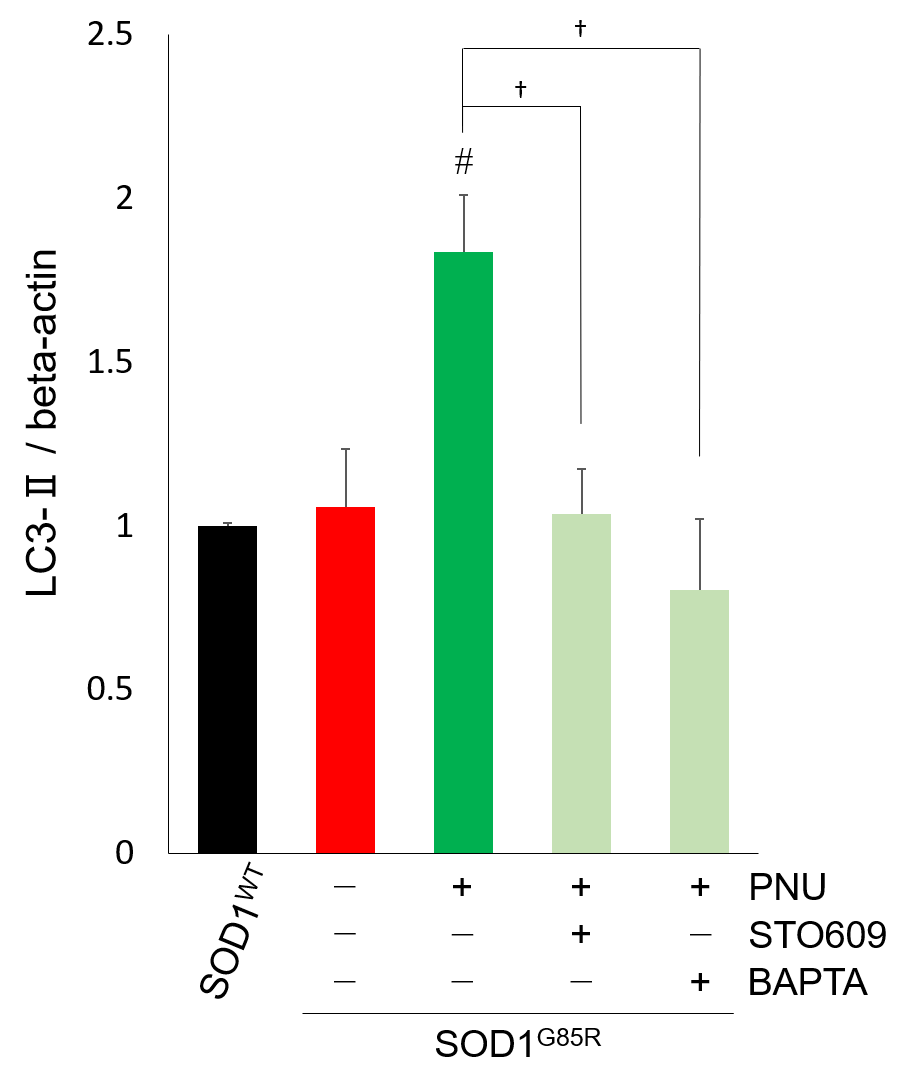


**Supplementary Figure 3.** (a, b) N2a cells expressing SOD1G85R were treated with 1 µM PNU282987 (PNU) in the presence or absence of 1 µM STO609 (STO) or 1 µM BAPTA-AM (BAPTA) after 24 h of transfection. The lysates were analyzed by immunoblotting with antibodies for LC-3. Relative levels normalized by the expression of beta-actin were quantified, based on the density of SOD1WT. (a) Corresponding to Fig 2B; (b) Corresponding to Fig 3B. Data is expressed as mean ± SEM from three independent experiments. Significance: # p < 0.05, ## p < 0.01 vs. SOD1G85R; † p < 0.05 vs SOD1G85R with PNU282987.

**(a)**


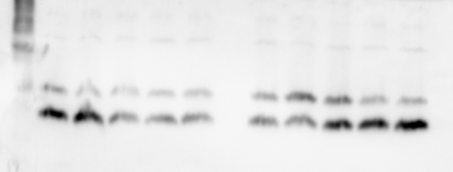


**LC3**

15 kDa

15kDa

20kDa

30kDa

**(b)**


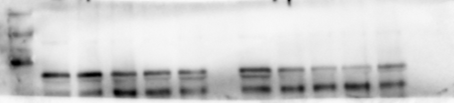


**p62**

62 kDa

70kDa

100kDa

140kDa

**(c)**


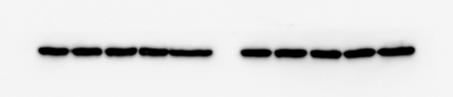


**beta-actin**

43 kDa

40kDa

50kDa

**Supplementary Figure 4.** (a) Whole representative western blot of LC3; (b) Whole representative western blot of p62; (c) Whole representative western blot of beta-actin. The portion surrounded by a red square is presented in Fig. 2A.

**(a)**


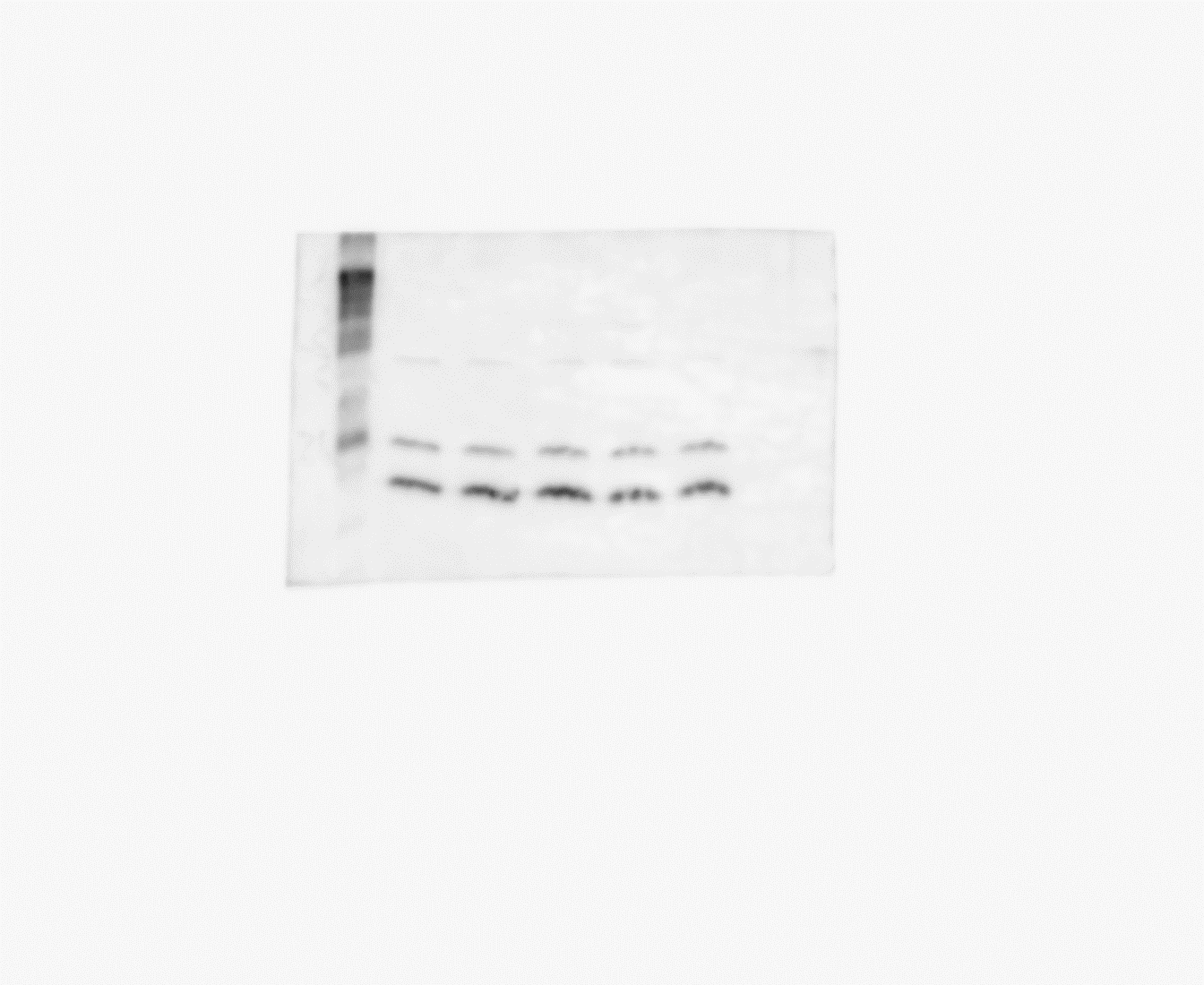


**LC3**

15 kDa

15kDa

20kDa

30kDa

**(b)**


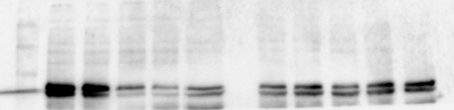


**p62**

62 kDa

70 kDa

100 kDa

140 kDa

**(C)**


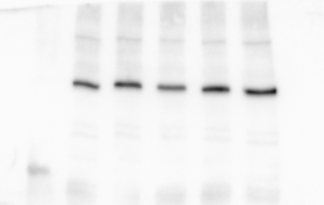


**p-mTOR**

289 kDa

140 kDa

**(d)**


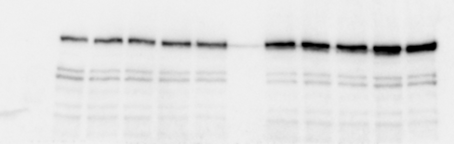


**mTOR**

289 kDa

140 kDa

**(e)**


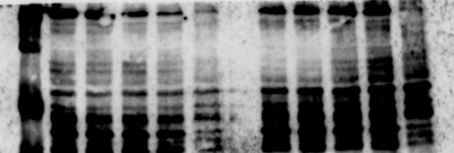


**p-AMPK**

62 kDa

50 kDa

70 kDa

100 kDa

**(f)**


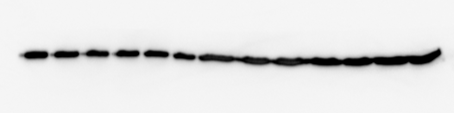


**beta-actin**

43 kDa

40 kDa

50 kDa

**Supplementary Figure 5.** (a) Whole representative western blot of LC3; (b) Whole representative western blot of p62; (c) Whole representative western blot of p-mTOR; (d) Whole representative western blot of mTOR; (e) Whole representative western blot of p-AMPK; (f) Whole representative western blot of beta-actin. The portion surrounded by a red square is presented in Fig. 3A.

**(a)**


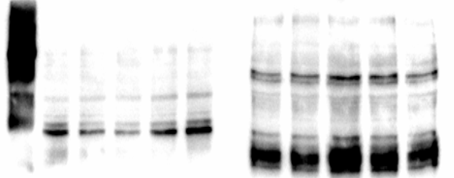


**TFEB**

66 kDa

70 kDa

100 kDa

140 kDa

**(b)**


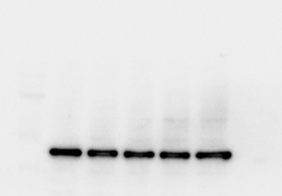


**GAPDH**

37 kDa

40 kDa

70 kDa

30 kDa

50 kDa

**(c)**


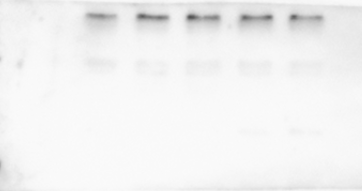


**Histone H3**

17 kDa

15 kDa

**(d)**


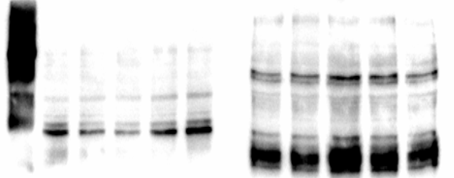


**TFEB**

66 kDa

70 kDa

100 kDa

140 kDa

**(e)**


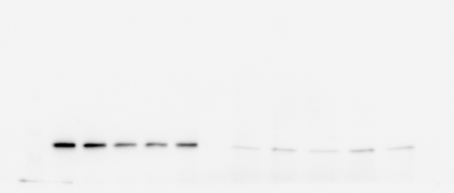


**GAPDH**

37 kDa

40 kDa

70 kDa

30 kDa

50 kDa

**(f)**


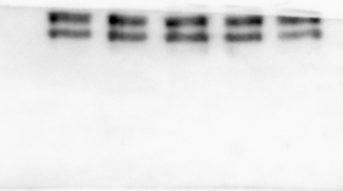


**Histone H3**

17 kDa

15 kDa

**Supplementary Figure 6.** (a) Whole representative western blot of TFEB in cytoplasmic fractions; (b) Whole representative western blot of GAPDH in cytoplasmic fractions; (c) Whole representative western blot of Histone H3 in cytoplasmic fractions; (d) Whole representative western blot of TFEB in nuclear fractions; (e) Whole representative western blot of GAPDH in nuclear fractions; (f) Whole representative western blot of Histone H3 in nuclear fractions. The portion surrounded by a red square is presented in Fig. 5B.
